# Efficient Harvesting of Irregular and Low‐Frequency Mechanical Energy via Hybridized Electromagnetic‐Triboelectric Systems

**DOI:** 10.1002/smll.202514996

**Published:** 2026-04-11

**Authors:** Yongbin Han, Ju‐Hyuck Lee, Hong‐Joon Yoon

**Affiliations:** ^1^ Department of Semiconductor Engineering Gachon University Seongnam‐si Gyeonggi‐do Republic of Korea; ^2^ Department of Energy Science and Engineering Daegu Gyeongbuk Institute of Science and Technology (DGIST) Daegu Republic of Korea

**Keywords:** electromagnetic generators, hybrid energy harvesters, renewable energy sources, self−powered sensors, triboelectric nanogenerators

## Abstract

The rapid expansion of the Internet of Things (IoT) and wearable electronics necessitates sustainable power sources to replace the limited electrochemical battery. While mechanical energy harvesting offers a promising solution, traditional harvesters face challenges of irregular and low−frequency environmental sources. This review presents the hybridized electromagnetic‐triboelectric nanogenerator (HE−TENG) as an effective strategy to overcome individual limitations of the electromagnetic generator (EMG) and triboelectric nanogenerator (TENG). By integrating the high−current characteristics of the EMG with the high−voltage, low−frequency efficiency of the TENG, the HE−TENG achieves a complementary broadband frequency response and enhanced energy conversion efficiency. The fundamental working principles, theoretical models, and impedance matching requirements of both mechanisms are analyzed to elucidate their synergistic coupling. Furthermore, recent advances in structural design are categorized based on diverse mechanical energy sources, including wind, water waves, and hydrokinetic flows. The analysis highlights specific hybridization strategies, such as bio−inspired mechanisms and variable transmission systems, to optimize performance under stochastic environmental conditions.

## Abstract 2

This review highlights the hybridized electromagnetic‐triboelectric nanogenerator (HE‐TENG) as a robust paradigm for scavenging irregular, low‐frequency mechanical energy. By integrating structural optimization with electrical impedance‐matching strategies, HE‐TENGs achieve a complementary broadband response. Recent advancements across diverse energy sources, such as wind, blue energy, vibrations, and human biomechanics, are systematically analyzed through an integrated synergy framework. Finally, critical technical bottlenecks and future perspectives are discussed to facilitate the realization of autonomous, maintenance‐free IoT networks.

## 1 Introduction

**FIGURE 1:**
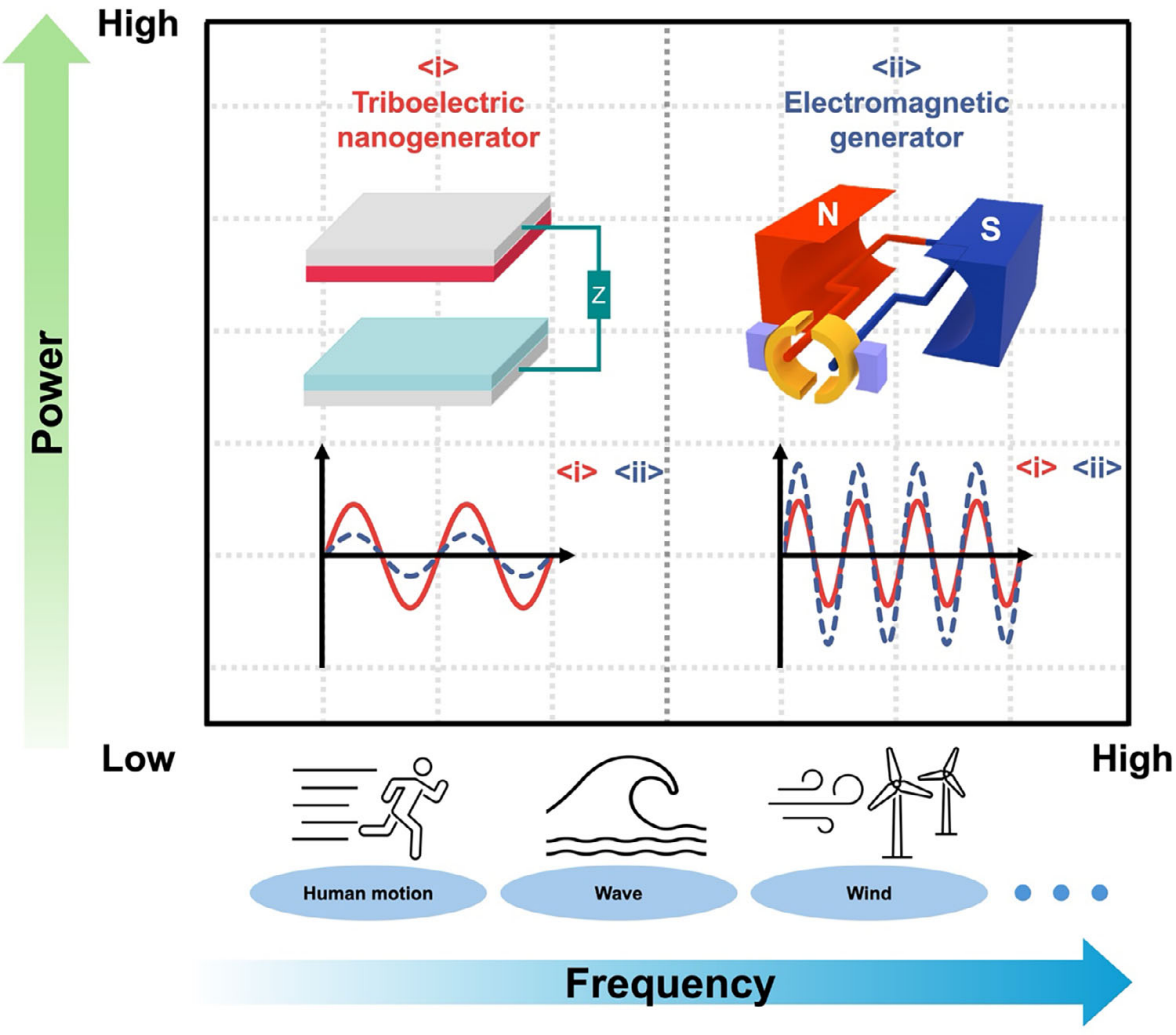
Schematic of the power generation characteristics of triboelectric and electromagnetic generators across different frequency ranges of mechanical energy sources.

In modern society, energy serves as the fundamental cornerstone of economic development and quality of life, leading to ever−increasing global demand [1]. Currently, global energy supply relies mostly on fossil fuels that are non−renewable energy sources, like coal, oil, and natural gas, and pose significant environmental concerns that include pollution, greenhouse gas emissions, and climate change [2, 3]. However, in the era of the Internet of Things (IoT), Artificial intelligence (AI), and big data, the widespread use of energy through portable devices, like wearables [4, 5] and implantable devices [6, 7], has led to a rapid increase in resource consumption, and demand for continuous and independent power sources [8]. Traditionally, these devices have relied on electrochemical batteries. However, batteries face critical limitations, such as finite lifespan, the need for periodic replacement or recharging, bulky size that hinders device miniaturization, and potential environmental hazards upon disposal. Consequently, developing sustainable, maintenance−free, and self−powered systems has become a critical challenge to ensure the long−term and stable operation of the next−generation electronics network. To resolve the challenges of energy consumption, active research is being conducted on renewable energy sources, such as the solar cell [9, 10], thermal energy [11, 12], and mechanical energy [13−15]. The solar cell is limited by weather conditions, day‐night cycles, and the inability to function effectively indoors or in shaded areas. Thermal energy harvesters, which rely on the Seebeck effect, require a substantial temperature gradient, which due to thermal equilibrium is often difficult to maintain in ambient environments, or on the human body. In contrast, mechanical energy is available in various forms, including wind [16, 17], waves [18, 19], water flow [20, 21], human motion [22, 23], and vibration [24, 25]. Mechanical energy harvesters have been investigated to transform mechanical energy into electrical energy, with primary mechanisms that include piezoelectric [26−29], electromagnetic [30−32], electrostatic induction [33, 34], and triboelectrification [35, 36]. The energy generated by such harvesters can be rectified and stored in rechargeable batteries [37, 38] or supercapacitors [39, 40], and subsequently used to supply power electronic devices on demand. Among the diverse energy scavenging mechanisms, triboelectric nanogenerators (TENGs) [41] and electromagnetic generators (EMGs) [42] have emerged as the most prominent paradigms for macroscopic mechanical energy harvesting. Nevertheless, practical energy extraction from real world environments poses a critical challenge, given that ambient mechanical stimuli, such as natural wind, ocean waves, and human biomechanics, are intrinsically irregular, highly stochastic, and predominantly low‐frequency. When employed independently for such environments, TENGs and EMGs present contrasting merits and inherent limitations. TENGs exhibit exceptional sensitivity to small amplitude, low‐frequency mechanical triggers, yielding high voltage outputs; however, their performance is fundamentally constrained by low output currents, high internal impedance, and progressive mechanical wear at frictional interfaces. Conversely, EMGs deliver robust, high current outputs and operate with high efficiency under continuous, high‐frequency excitations, yet their energy conversion efficiency deteriorates precipitously in response to low‐frequency and stochastic inputs. To circumvent these inherent constraints, extensive single device optimizations have been pursued. For TENGs, approaches including surface texturing, structural engineering, and charge injection have been employed to maximize surface charge density, while non‐contact or rolling architectures have been introduced to alleviate interfacial wear and prolong operational lifespan. Parallelly, EMG optimizations have heavily relied on mechanical frequency up‐converters (e.g., gear trains or mechanical springs) and magnetic flux concentrators to enhance responsiveness to low‐frequency stimuli and maximize magnetic flux gradients. Despite these notable advancements, singular harvesting modules are fundamentally inadequate for realistic, irregular natural environments [43, 44, 45, 46]. TENGs are still limited by high internal impedance and charge dissipation, whereas EMGs remain practically ineffective in ultra low frequency and highly stochastic regimes. As systematically demonstrated by Zhao et al. [47]., TENGs possess a distinct superiority in energy conversion efficiency over EMGs in the low‐frequency domain (typically < 5 Hz) and under small‐amplitude excitations. Because practical ambient energy sources fluctuate randomly and exhibit broad, low‐frequency spectra, relying on solitary generator technology is intrinsically suboptimal. Consequently, the structural and electrical hybridization of EMGs and TENGs (HE‐TENGs) [48] represents not merely an optional augmentation, but an essential paradigm for realizing complementary, broadband energy harvesting systems. Although previous comprehensive reviews [49, 50, 51, 52] have addressed HE‐TENGs and broader TENG‐based hybrid systems, they have predominantly classified devices based on physical architectures (e.g., rotational, sliding, pendulum, and cantilever modes) or broadly surveyed their application domains, including wearables, bioelectronics, and blue energy. A systematic framework that examines the integrated coupling of EMGs and TENGs—specifically focusing on the resolution of intrinsic impedance mismatches and adaptation to stochastic, low‐frequency ambient sources—has not been addressed. To address this research gap, this review introduces a comprehensive integration framework for HE‐TENGs that addresses both electrical considerations (impedance matching, power management, and output coupling) and mechanical strategies (structural design, bandwidth broadening, and dynamic mode transition). Recent advances in HE‐TENG technologies are systematically classified and analyzed according to the dynamic profiles of stochastic mechanical sources, including wind, water waves, hydrokinetic flows, human biomechanics, and structural vibrations. This perspective demonstrates how the proposed integration framework is applied to address the distinct behaviors and spectral complexities inherent to each energy environment. Furthermore, this review evaluates representative implementations of HE‐TENGs that power low‐power sensors, wireless communication modules, and IoT‐integrated electronics. This assessment provides essential design guidelines and formulates research directions for reliable,.broadband hybrid energy‐harvesting systems (Figure 1).

## 2 Working Mechanisms and Electrical Characteristics

### 2.1 Operating Principle and Transduction Mechanism of the Electromagnetic Generator

**FIGURE 2:**
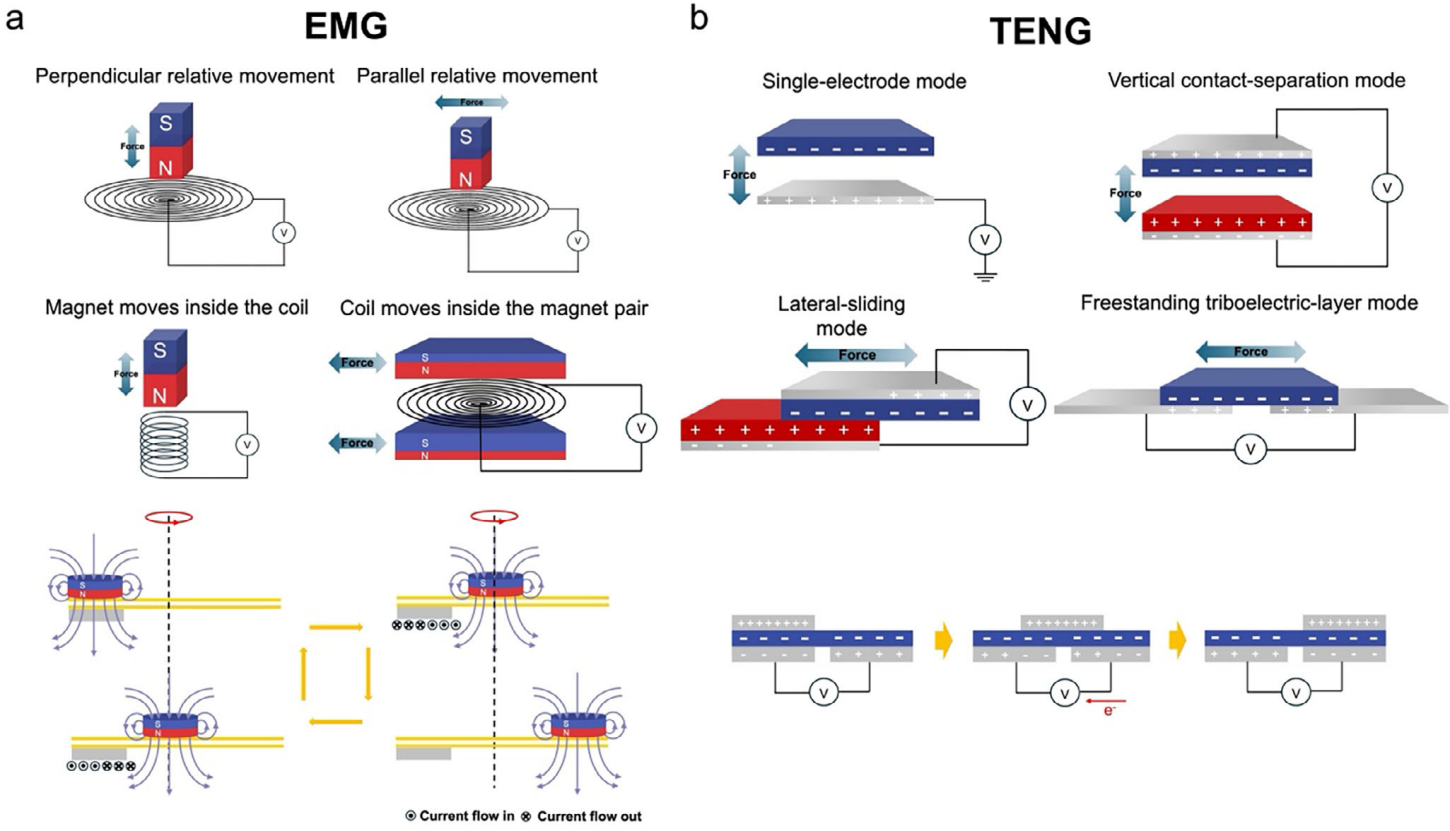
(a) and (b) Schematics of the four basic working modes and working principles of the EMG and TENG.

The operating principle of the EMG is based on Faraday's law of electromagnetic induction, where the movement between a magnet and a coil induces electricity by changing the magnetic flux over time within the coil. In contrast, the TENG [53] generates charge through friction, arising from the coupling of triboelectrification between materials with different surface charges and electrostatic induction [54]. These mechanisms enable operation in a variety of modes. Figure 2a illustrates the four basic operating modes of the conventional EMG: perpendicular relative movement, parallel relative movement, the magnet moving inside the coil, and the coil moving inside the magnet pair. The induced electromotive force (*ε*) of such an EMG can be expressed as:

(1) $$\varepsilon =-N\cdot \;\frac{d\Phi }{dt}=-N\cdot \frac{dB}{dt}\cdot S$$

where, *N* is the number of coil turns, d*Φ*/d*t* is the rate of change of magnetic flux, d*B*/d*t* is the rate of change of the magnetic field, and *S* is the area of the coil. This equation shows that the induced electromotive force is determined by the number of turns and area of the coil (larger values resulting in higher output), as well as the rate of change of the magnetic field (which increases with stronger magnetic materials and higher speeds driven by mechanical energy).

### 2.2 Operating Principle and Transduction Mechanism of the Triboelectric Nanogenerator

Unlike the EMG, the TENG converts mechanical energy into electrical energy through the coupling effects of triboelectrification and electrostatic induction [33−36]. When two materials with different electron affinities come into contact, they generate triboelectric charges of opposite polarity. Subsequent separation of the materials induces a potential difference between the electrodes, thereby driving an electron flow through an external circuit. Figure 2b illustrates the four basic operating modes of the TENG: single−electrode mode [55], lateral−sliding mode [56], vertical contact‐separation mode [57], and freestanding mode [58]. The output characteristics of a TENG can be generally described as follows [59]:

(2) $$\;V_{\mathrm{out}}=-\frac{1}{C\left(x\right)}Q+V_{OC}\left(x\right)$$

where, *V*_OC_ is the open circuit voltage, *C*(*x*) is the capacitance between the electrodes, and *Q* is the transferred charges between electrodes. The voltage expressions for each mode are as follows. For the single−electrode mode TENG, the voltage can be expressed as [60]:

(3) $$\;V_{\;\mathrm{OC}}^{S}=\frac{\sigma S}{2C_{0}}$$

where, *S* is the electrode area, *C*_0_ is the capacitance between the main electrode and reference electrode, and σ is the surface charge density. For the vertical contact‐separation mode TENG, the voltage can be expressed as [61]:

(4) $$\;V_{\;\mathrm{OC}}^{\mathrm{CS}}=\frac{\sigma x}{\varepsilon_{0}}$$

where, *x* is the distance between the two triboelectric layers, and ε_0_ is the permittivity of free space. For the lateral−sliding mode TENG, the voltage can be expressed as [62]:

(5) $$\;V_{\;\mathrm{OC}}^{L}=\frac{\sigma xd_{0}}{\varepsilon_{r}\varepsilon_{0}\left(l-x\right)}$$

where, *d*_0_ is the effective thickness constant, ε_*r*_ is the permittivity of triboelectric material, and *l* is the length of plate. For the Freestanding mode TENG, the voltage can be expressed as [63]:

(6) $$\;V_{\;\mathrm{OC}}^{f}=\frac{\sigma S}{C}$$

where, *C* is the capacitance between the two electrodes. These equations indicate that the output performance of the TENG is primarily dictated by the properties of the triboelectric materials. Therefore, the TENG utilizes various conventional materials, and can be fabricated to be mechanically flexible and lightweight [64].

### 2.3 Complementary Electrical Characteristics and Hybridization Mechanism

**FIGURE 3:**
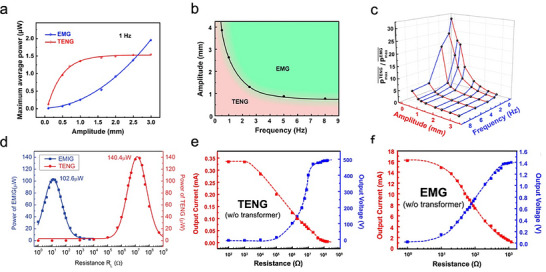
Comparison of the EMG and TENG characteristics. a) Maximum average power comparison of the EMG and TENG vs. amplitude at 1 Hz. b) Domain of excitation amplitude and frequency values, including the amplitudes at crossover points of the two characteristic curves at different frequencies, where the energy harvesting performance of the test EMG or TENG is superior. The light red area denotes the dominant scope of the TENG in low−frequency and small−amplitude, while the light green area denotes that of the EMG. c) Maximum average output power ratio of the EMG and TENG vs. amplitude and frequency. (a, b, and c) Reproduced with permission [47]. Copyright 2019, Elsevier. d) Output power of the EMG and TENG as a function of different load resistances. Reproduced with permission [54]. Copyright 2014, Wiley‐VCH. (e and f) Voltage and current output of the TENG and EMG, respectively, for different loads. Reproduced with permission [65]. Copyright 2019, Elsevier.

Zhao et al. [47] investigated the influence of motion amplitude and frequency on the conversion mechanisms of the EMG and TENG. They reported that at an environmental frequency of 1 Hz with small amplitudes (below 2.5 mm), the TENG can generate a higher maximum average power than the EMG (Figure 3a). Furthermore, compared to the EMG, the TENG maintains a relatively stable maximum average power above a specific amplitude (≥ 1 mm), demonstrating its ability to achieve more stable energy conversion from irregular mechanical sources. Figure 3b summarizes the amplitudes at which the maximum average power characteristics of the two generators intersect at various frequencies, indicating that TENG outperforms EMG in the low−frequency and small−amplitude range. Furthermore, Figure 3c presents the ratio of the maximum average power between TENG and EMG as a function of amplitude and frequency, exhibiting a decreasing tendency with increasing amplitude and frequency. This result suggests that the TENG provides distinct advantages over the EMG, particularly in the low−amplitude and low−frequency regions.

To maximize the electrical performance of the TENG and EMG, different impedance requirements must be satisfied (Figure 3d). Specifically, when the external load impedance is significantly smaller than the internal impedance, a TENG can be regarded as a current source with large internal impedance. In contrast, when the external load impedance is considerably larger than the internal impedance, an EMG can be regarded as a voltage source with small internal impedance. Despite these complementary output characteristics, the direct integration of TENG and EMG is fundamentally hindered by a severe impedance mismatch. TENGs inherently possess a high internal impedance (MΩ to GΩ) and deliver high voltage, low current output, whereas EMGs exhibit a low internal impedance (Ω to kΩ) and behave as low voltage, high current sources (Figure 3e,f). Therefore, simply combining them without a strategic design is insufficient; a systematic impedance‐matching framework is required to mitigate the internal power dissipation caused by this extreme gap. At the circuit level, bridging this extreme impedance gap necessitates a dedicated power‑management interface that integrates tailored rectifiers, intermediate storage capacitors, and impedance‑transforming converters (e.g., buck or resonant converters), thereby matching the disparate source impedances and minimizing internal power loss [12, 41]. Such synergistic power integration ensures that the high voltage pulses from the TENG and the continuous high current flow from the EMG are rectified and regulated into a unified DC bus with minimal loss under fluctuating inputs. Furthermore, this hybridization utilizes structural integration to mitigate inherent mechanical constraints, establishing a unified architectural framework that achieves substantial volumetric efficiency and weight reduction compared to discrete devices. More importantly, this mechanical coupling ensures that a single kinematic source, irrespective of its motion characteristics (rotational or reciprocating), to simultaneously drive both the electromagnetic and triboelectric mechanisms. Such integration addresses the inherent limitations of standalone generators, which establishes HE‐TENG architecture as a versatile strategy for the broadband scavenging of irregular mechanical energy. Table 1 contrasts the mechanical and electrical characteristics of TENGs and EMGs, identifying the fundamental performance mismatches that justify the hybrid integration strategies analyzed in this review. Utilizing these complementary characteristics, recent research [66, 67, 68, 69] has established integrated HE‐TENG architectures that effectively scavenge energy from diverse environmental sources.

**TABLE 1:** Summarized overall comparison table between the complementary characteristics of the EMGs and TENGs.

	TENG	EMG
Working Mechanism	Triboelectrification and Electrostatic Induction	Electromagnetic Induction
Open−circuit Voltage	High	Low
Short−circuit Current	Low	High
Output Power	Low	High
Working Frequency	Low	High
Power Characteristic	AC	AC
Internal Impedance	Large	Small
Amplitude	Small	Large

## 3 Development of HE−TENGs for Harvesting Various Mechanical Energy Sources

### 3.1 Wind Energy Harvesting

Wind energy is a widely accessible renewable resource in nature; however, its inherent intermittency and fluctuation present significant challenges for consistent energy conversion, particularly under varying wind velocities [70]. While the traditional EMG demonstrates superior performance at high wind velocities, its efficiency drops notably in low−velocity conditions [71]. In contrast, the TENG differs by effectively harvesting energy even from low−velocity wind [72], thereby enabling efficient operation across a wider spectrum. Consequently, the integration of the EMG and TENG into a hybrid system capitalizes the synergistic advantages of both technologies, ensuring stable and efficient energy harvesting across diverse wind environments.

**FIGURE 4:**
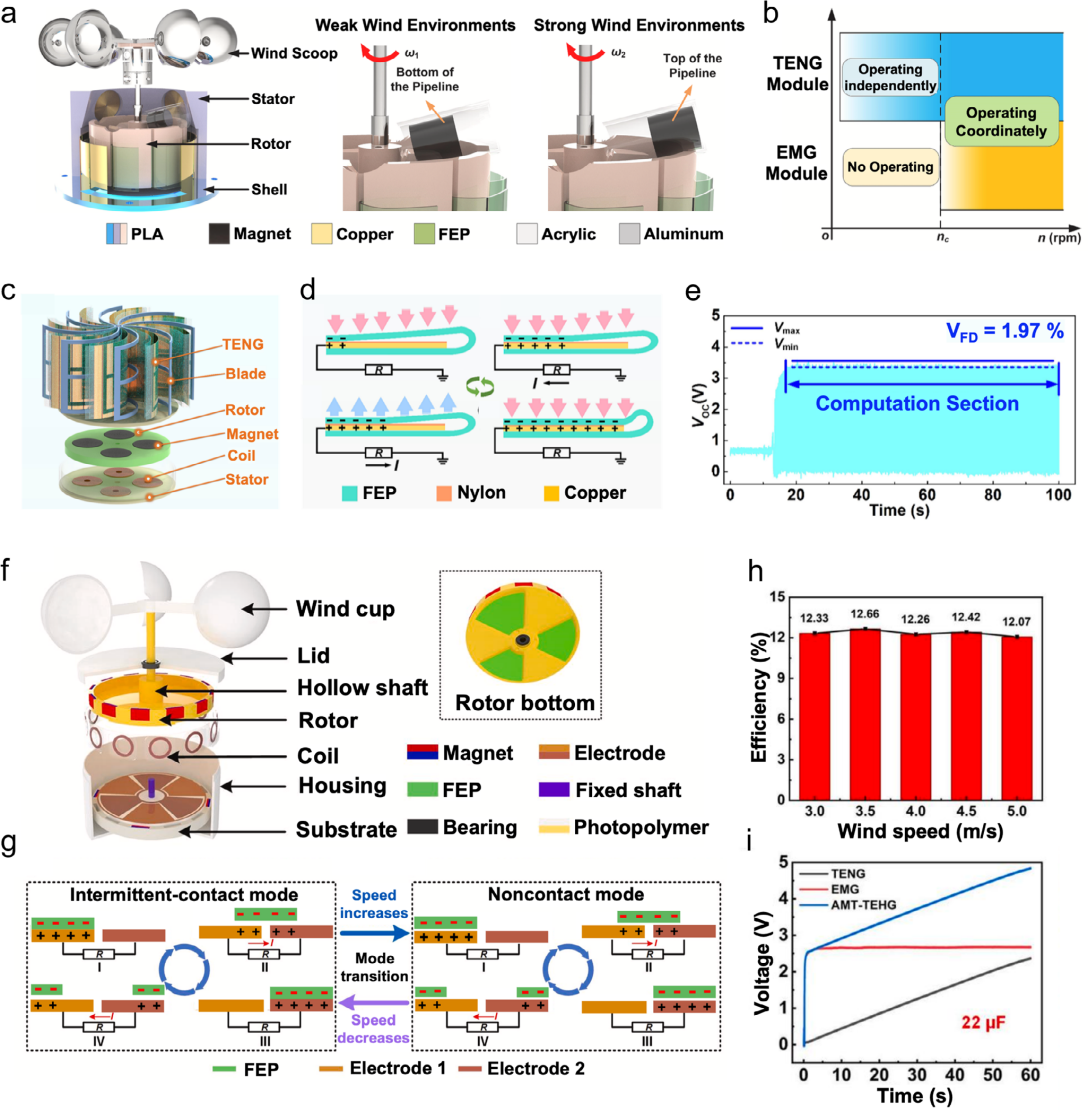
Hybrid HE−TENG for efficient energy harvesting from wind energy. a) Schematic and working principle of the FC−TEH in different wind speed environments. b) The operating points of the TENG and EMG under different wind environments. Reproduced with permission [73]. Copyright 2022, Elsevier. c) Structure of the SO−TEHG, and d) Power generation principle of the TENG unit. e) Voltage output under random wind energy. Reproduced with permission [74]. Copyright 2024, ACS Publications. f) Structure and components of the AMT−TEHG. g) The triboelectrification process in the IC−mode and electrostatic induction in the NC−mode. h) Energy Conversion Efficiency of the AMT−TEHG under various wind velocities. i) Voltage curves of a 22 µF capacitor charged by the TENG unit, EMG unit, and AMT−TEHG. Reproduced with permission [75]. Copyright 2022, Elsevier.

X. Li et al. proposed a flexible cooperative triboelectric−electromagnetic harvester (FC−TEH) designed to optimize wind energy harvesting across varying wind velocities [73]. In this system, the TENG module operates efficiently at low wind velocities, while the EMG module is activated as wind velocity increases, enhancing overall energy conversion efficiency. Figure 4a illustrates the structure of the FC−TEH. The operational mechanism of the FC−TEH adapts according to the ambient wind velocity. Under low−velocity conditions, weak airflow causes the rotor structure to rotate slowly, activating the sliding−mode TENG, where fluorinated ethylene propylene (FEP) films slide against copper electrodes to generate electricity. At this stage, the incident wind velocity remains below the critical threshold, preventing the EMG module from operating. Specifically, the magnet within the EMG module is constrained by friction with the pipeline, which restricts its movement and maintains a separation distance from the coil. Consequently, electromagnetic induction remains negligible, leading to minimal power generation. As the wind velocity increases, the magnet gradually overcomes the frictional force and translates toward the top of the pipeline, decreasing the gap between the magnet and coil. This enhanced proximity strengthens the electromagnetic induction, allowing the EMG module to efficiently convert high−velocity wind energy into electrical power (Figure 4b). The FC−TEH is activated at a wind velocity of 4 m/s and reaches its critical operational velocity at 6 m/s. The system generates a maximum output power of 20 mW at a natural wind velocity of 8 m/s. This configuration enables the selective operation of the generator based on wind velocity, where below a specific threshold, the TENG operates independently, and above this threshold, both TENG and EMG function simultaneously. Although this configuration may yield lower power generation compared to other HE−TENG systems where both units operate simultaneously at lower velocities, it demonstrates the system's potential as a self−powered sensor to distinguish wind velocities relative to a specific threshold. From a structural perspective, the FC‐TEH utilizes the low startup torque of the TENG‐only mode under weak wind conditions, which facilitates rotation initiation at lower velocities. The EMG component is activated only after the rotor velocity surpasses a critical threshold, ensuring that the structural dynamics are synchronized with the stochastic distribution of ambient wind velocities. From an electrical perspective, this velocity‐dependent mode switching establishes a partitioned operation where the high‐impedance TENG governs the low‐speed regime. Conversely, the low‐impedance EMG activates at higher velocities to augment power output, which ensures optimized electrical conversion across a broad spectral range. Furthermore, the nonlinear transition in EMG output near the threshold enables the FC‐TEH to function as a self‐powered wind speed sensor that provides autonomous monitoring in stochastic natural environments. In this study, Y. Wang et al. [74] proposed a steady−output triboelectric−electromagnetic hybrid generator (SO−TEHG) incorporating variable drag turbine blades to mitigate output fluctuations and address the high cut−in wind velocity issues in wind energy harvesting. Figure 4c shows the SO−TEHG. The TENG module of the SO−TEHG is designed as a variable drag turbine blade, utilizing materials such as FEP, nylon, and copper, with flaps attached to the trailing edge to enhance aerodynamic efficiency. As wind pressure induces contact between the FEP and nylon layers, charge transfer occurs, establishing an electrostatic potential and generating electrical power (Figure 4d). This design can generate a voltage of 50 V even at a wind velocity of 2 m/s, with a peak output of 0.8 mW. The SO−TEHG integrates a voltage and current stability module to minimize voltage fluctuation (*V*_FD_) and current fluctuation (*I*_FD_), ensuring consistent power output under irregular wind conditions. Figure 4e shows that the *V_FD_* of the SO−TEHG is 1.97%, indicating stable output even under fluctuating wind velocities. This stability is achieved by regulating the output discrepancy between low and high wind velocities, thereby attenuating fluctuations and maintaining a steady power supply. In terms of mechanical design, the variable drag turbine blades utilize the structural flexibility of the TENG unit to lower the cut‐in wind speed relative to conventional EMG‐based harvesters. This improved aerodynamic response under low‐velocity regimes is essential for capturing low‐frequency and low‐amplitude wind excitations. From an electrical perspective, the integrated power management circuit stabilizes the stochastic fluctuations of TENG and EMG outputs through the regulation of rectified voltage and current. This configuration maintains minimal *V_FD_* and *I_FD_* values even as ambient wind speeds vary stochastically between 1 and 10 m/s. The coordination between aerodynamic optimization and power conditioning illustrates how mechanical and electrical subsystems are co‐engineered to provide a stable power supply for remote sensing and IoT applications. In traditional wind energy harvesters, the relatively high cut−in wind velocity required for EMGs limits efficiency. Furthermore, for TENGs, continuous sliding friction leads to material abrasion and energy dissipation. To address these challenges, K. Fan et al. [75] designed the Automatic−mode−transition rotary triboelectric hybrid nanogenerator (AMT−TEHG) shown in Figure 4f. This generator automatically transitions between intermittent−contact (IC) mode and non−contact (NC) mode based on wind velocity. Figure 4g shows that utilizing a magnetic levitation (Maglev) mechanism, intermittent sliding contact occurs between the film and the electrodes at a low wind velocity of 2.4 m/s. This allows the surface charges of the film to be replenished with low frictional resistance, significantly reducing material abrasion, which also contributes to a reduction in the cut−in wind velocity. As the wind velocity gradually increases and reaches 3.0 m/s, the device transitions to NC mode, generating electrical output without frictional resistance between the film and electrodes. This mechanism ensures that the energy conversion efficiency remains substantially constant regardless of wind velocity variations. As confirmed by Figure 4h, the energy conversion efficiency remains nearly constant even as the wind velocity increases from 3.0 to 5.0 m/s. Furthermore, in the AMT−TEHG system, the rectified outputs from the two units are connected in parallel for accumulation in a capacitor, as shown in Figure 4i. While the EMGs are characterized by high current and low voltage, enabling rapid charging but limited by low saturation voltage, the TENGs exhibit high voltage characteristics. By hybridizing these complementary outputs, the AMT−TEHG simultaneously achieves both rapid charging capability and high saturation voltage. This study partially overcomes the fundamental issue of material abrasion caused by friction in TENGs. Although a voltage drop occurs during the transition from IC mode to NC mode as the wind velocity increases, the system demonstrates significant potential for applications requiring long−term durability and operation. From a mechanical perspective, the AMT‐TEHG utilizes maglev‐mediated vertical oscillation of the rotor to enable an autonomous transition from intermittent contact (IC) to non‐contact (NC) mode as wind velocity increases. This mechanism addresses the inherent tradeoff between effective surface charge replenishment at low speeds and mechanical durability at high speeds. From an electrical perspective, the parallel rectification of TENG and EMG outputs synthesizes the high‐voltage profile of the TENG with the high‐current density of the EMG into a single storage capacitor, ensuring both high saturation voltage and rapid charge accumulation under low‐velocity regimes. Consequently, the AMT‐TEHG demonstrates that engineered mechanical mode transitions, coupled with parallel output integration, can effectively mitigate material abrasion, lower cut‐in wind speeds, and maintain stable energy conversion efficiency across a broad spectrum of stochastic wind profiles.

### 3.2 Development of HE−TENGs for Hydrokinetic Energy Harvesting

Water is a ubiquitous natural resource and an abundant source of clean renewable energy with immense potential, encompassing forms such as waves, tides, thermal gradients, salinity gradients, and ocean currents [76]. Consequently, extensive research on energy harvesting from aquatic environments is being actively pursued [77, 78, 79]. While EMG is the conventional standard for harvesting hydrokinetic energy, it often necessitates complex mechanical structures and hydraulic systems [18]. Furthermore, the inherent low−frequency and low−amplitude characteristics of fluid motions are unfavorable for the efficient operation of the EMG, necessitating the supplementation of alternative technologies. In contrast, the TENG demonstrates superior efficiency in harvesting low−frequency and low−amplitude mechanical energy, offering distinct advantages, which include simple structure [80], lightweight design [81], and high power density [82]. These attributes make the TENG highly suitable for hybridization with the EMG. This HE−TENG system not only broadens the operational frequency bandwidth for energy harvesting but also enhances the overall energy conversion efficiency. This section highlights recent studies focused on the effective harvesting of water flow and wave energy utilizing these hybrid systems.

#### 3.2.1 Wave Energy Harvesting

Wave energy holds significant promise as a renewable resource due to its high energy density and widespread accessibility [83]. However, the efficient extraction of wave energy remains a technological challenge. Traditionally, wave energy harvesting has relied on the EMG, which is often characterized by its bulky size, low efficiency at low frequencies, high construction costs, and complex mechanical design, resulting in inherent operational limitations. Conversely, the TENG offers compelling advantages, including cost−effectiveness, structural simplicity, lightweight form factor, and effectiveness in harvesting random, low−frequency excitations. Leveraging these attributes, numerous researchers are investigating hybrid generators that combine the EMG and TENG to achieve superior output performance.

**FIGURE 5:**
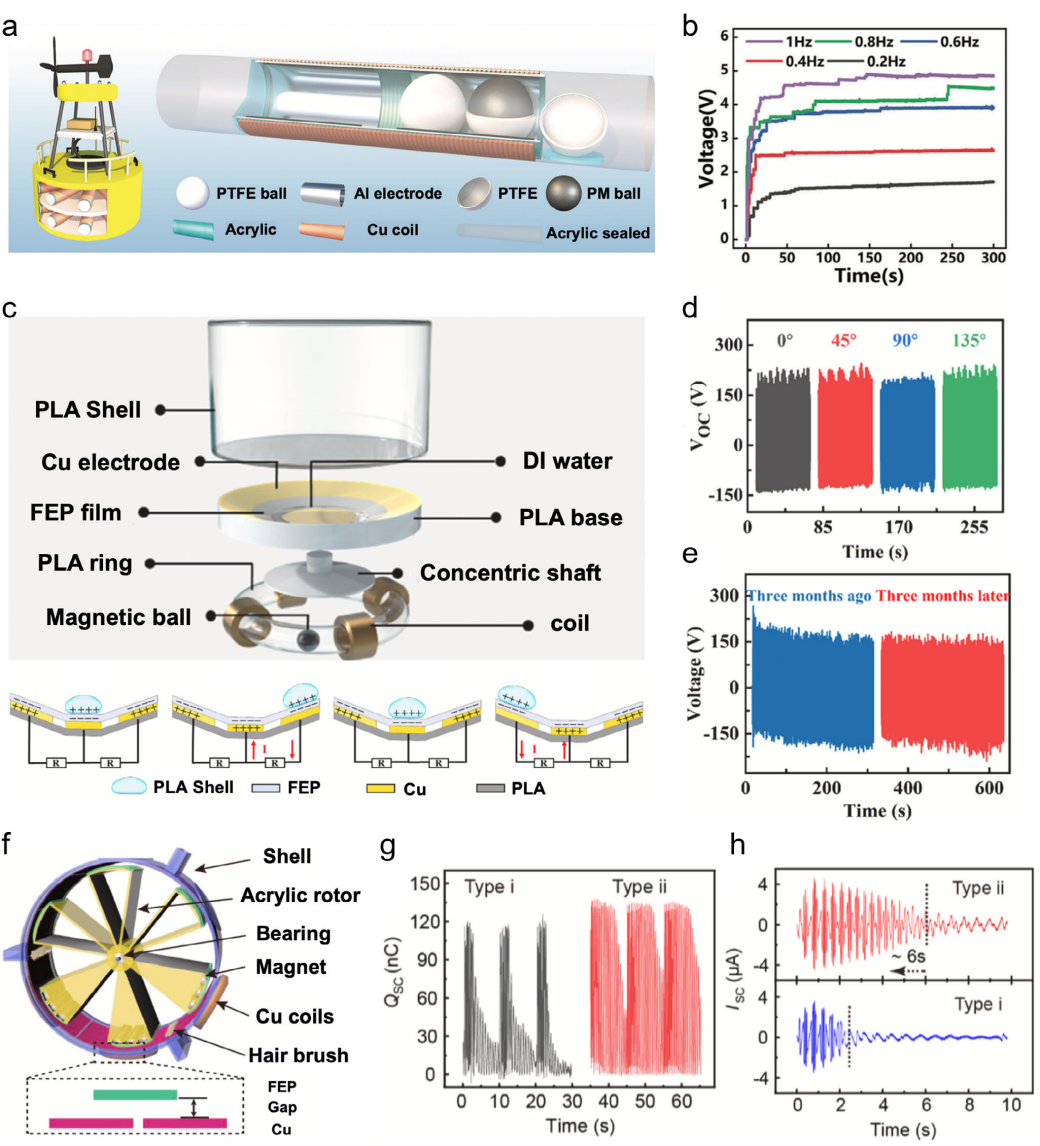
Hybrid HE−TENG for efficient energy harvesting from water wave energy. a) Schematic of the TEWEH integrated inside a buoy as the power supply, showing the internal view of the TEWEH structure. b) Charging a 330 µF capacitor using the TEWEH at different swinging frequencies. Reproduced with permission [84]. Copyright 2023, WILEY−VCH. c) Schematic of the iTEHG and working mechanism. d) Open−circuit voltage (*V*_OC_) from four orientations. e) *V*_OC_ before and after three months. Reproduced with permission [85]. Copyright 2023, Elsevier. f) Schematic of the hybrid nanogenerators for ocean wave energy harvesting showing an enlarged view of the gap between the FEP films and Cu electrodes. g) Comparison of *Q*_SC_ waveforms under one excitation for two blade types triggered by 0.1 Hz water waves. h) *I*_SC_ under one excitation for the two types of blades triggered by the water waves of 0.1 Hz. Reproduced with permission [86]. Copyright 2021, Elsevier.

C. Zhu et al. proposed a highly integrated triboelectric−electromagnetic wave energy harvester (TEWEH) that could scavenge wave energy over a wide frequency range and from random wave excitations, as shown in Figure 5a [84]. The TEWEH incorporates a permanent magnet (PM) ball embedded within a polytetrafluoroethylene (PTFE) shell to form a PM−PTFE sphere. This configuration integrates the magnetic component of the EMG and the dielectric material of the TENG, maximizing the volumetric efficiency of the harvester and significantly enhancing the output power density. The PM−PTFE ball responds to wave−induced vibrations, allowing both the TENG and EMG modules to operate simultaneously for electricity generation. The output performance of the constituent components was evaluated under horizontal and swinging excitations. Notably, the TEWEH demonstrated efficient wave energy harvesting capabilities even at low frequencies and amplitudes. Under conditions of 1 Hz and a ±30° swing angle, the output of the TENG component reached 230.25 V and 1.34 µA, while the average output of the EMG was 2.30 and 10.43 mA. Remarkably, even at an ultra−low frequency of 0.2 Hz, the system was able to charge a 330 µF capacitor (Figure 5b). This demonstrates the system's ability to efficiently harvest wave energy characterized by ultra−low frequencies and minute amplitudes. The integration of TENG and EMG components into a single unit via the innovative PM−PTFE ball module highlights the potential of the HE−TENG to operate synergistically as a unified system. Unlike traditional designs where TENG and EMG units function independently, this monolithic integration maximizes space utilization and facilitates simultaneous operation, thereby enhancing the overall energy harvesting efficiency. From a structural perspective, this high degree of integration exemplifies spatial synergy, wherein a single physical moving component (the PM‐PTFE ball) concurrently functions as both the magnetic flux source for the EMG and the dielectric friction layer for the TENG. From an electrical perspective, the system exhibits profound synergy by consolidating the high voltage TENG output with the high current EMG output. This configuration seamlessly overcomes impedance disparities, thereby facilitating the rapid charging of energy storage units for practical marine buoy applications. Q. Xu et al. proposed the guided liquid−based isotropic triboelectric−electromagnetic hybrid nanogenerator (iTEHG) to efficiently harvest energy, addressing the limitations of conventional TENGs, such as mechanical abrasion at solid−solid interfaces and directional dependence (Figure 5c) [85]. The iTEHG is constructed with a solid−liquid interface and a concentric circular electrode pair structure, based on a tilted satellite−dish−inspired substrate. This design enables enhanced regulation of liquid flow properties, improving isotropy compared to planar architectures that are inefficient under random and split flow characteristics. Consequently, the gravity−induced directional flow produces more stable and higher energy output under wave excitation. Given that ocean waves are multidirectional and irregular, the omnidirectional harvesting capability of the TENG was demonstrated by outputs from four directions: 0°, 45°, 90°, and 135° (Figure 5d). The relative standard deviation of both |*V*_avg_| and |*I*_avg_| from all directions was within 5%, indicating excellent isotropic performance. Thanks to the solid−liquid interface design and full encapsulation, the TENG module demonstrates high durability and resistance to humidity and corrosion, maintaining stable output over three months (Figure 5e). This confirms that the proposed solution effectively mitigates the issues of abrasion and directional dependence in the TENG, enabling efficient wave energy harvesting. From a structural perspective, this configuration demonstrates a hydrodynamic integration specifically engineered for marine environments. By utilizing the fluid‐structure interaction across a profiled unified substrate, the architecture effectively responds to the stochastic and omnidirectional characteristics of ocean waves. Consequently, this design enables the simultaneous operation of both transduction units via a direct‐drive mechanism, obviating the requirement for complex mechanical transmission systems. Y. Feng et al. developed a swing−structure−based hybrid nanogenerator utilizing a soft−contact cylindrical TENG (SCC−TENG) and an EMG, as showed in Figure 5f [86]. Incorporating flexible brush structures into the SCC−TENG significantly reduced the frictional resistance, improving device durability and mitigating charge leakage issues. Under wave excitation at 0.1 Hz, the peak power density was measured at 10.16 W m^−3^, with an average power density of 0.23 W m^−3^. The SCC−TENG experiments compared two brush types: a common fan−shaped blade (Type i) and a design with smooth edges in the chamfer regions (Type ii). The chamfered edges of Type ii enabled superior charging of the FEP film surface, significantly reducing operating resistance. Figure 5g shows that a greater charge transfer (*Q*_SC_ = 135 nC) was observed at 0.1 Hz, resulting in a higher peak short−circuit current (*I*_SC_ = 4.57 µA). Furthermore, as shown in Figure 5h, when the time required to pass at least one electrode area was defined as *τ*, the value for Type ii was 6.1 s, indicating superior operating conditions compared to Type i (*τ* = 2.4 s), which exhibited faster performance degradation. This study partially overcomes the fundamental challenge of low−frequency wave energy harvesting by optimizing the swing−based TENG and incorporating a modularized EMG. However, the system achieves maximum power at 1 Hz, with output decreasing as the frequency deviates. This behavior suggests that the system holds potential for applications in specialized environments characterized by a narrow frequency bandwidth, rather than in environments requiring broadband harvesting. Regarding the structural configuration, the shared swing architecture facilitates a mechanical coupling that enables a single ultra‐low‐frequency motion to simultaneously actuate both the soft‐contact TENG and the EMG units. Collectively, these investigations establish that structural and electrical hybridization represents an essential framework for addressing the ultra‐low‐frequency and multi‐directional constraints inherent to ocean wave energy harvesting.

#### 3.2.2 Water Flow Energy Harvesting

Water flow velocities in natural environments are typically low, averaging around 1 m s^−1^, which presents significant challenges for effective energy harvesting using traditional EMG−based systems [87]. The primary limitations of these conventional systems include insufficient output power and limited energy conversion efficiency, restricting their ability to efficiently capture and utilize energy from low−velocity water flows. Given that the TENG can effectively harvest and utilize irregular and low−frequency mechanical energy from fluid flow, extensive research is currently focused on hybrid generators combining TENG and EMG for use in water flow environments. However, further research remains essential to optimize the TENG and EMG for low−flow−velocity environments, ensuring their efficiency in capturing energy under such conditions.

**FIGURE 6:**
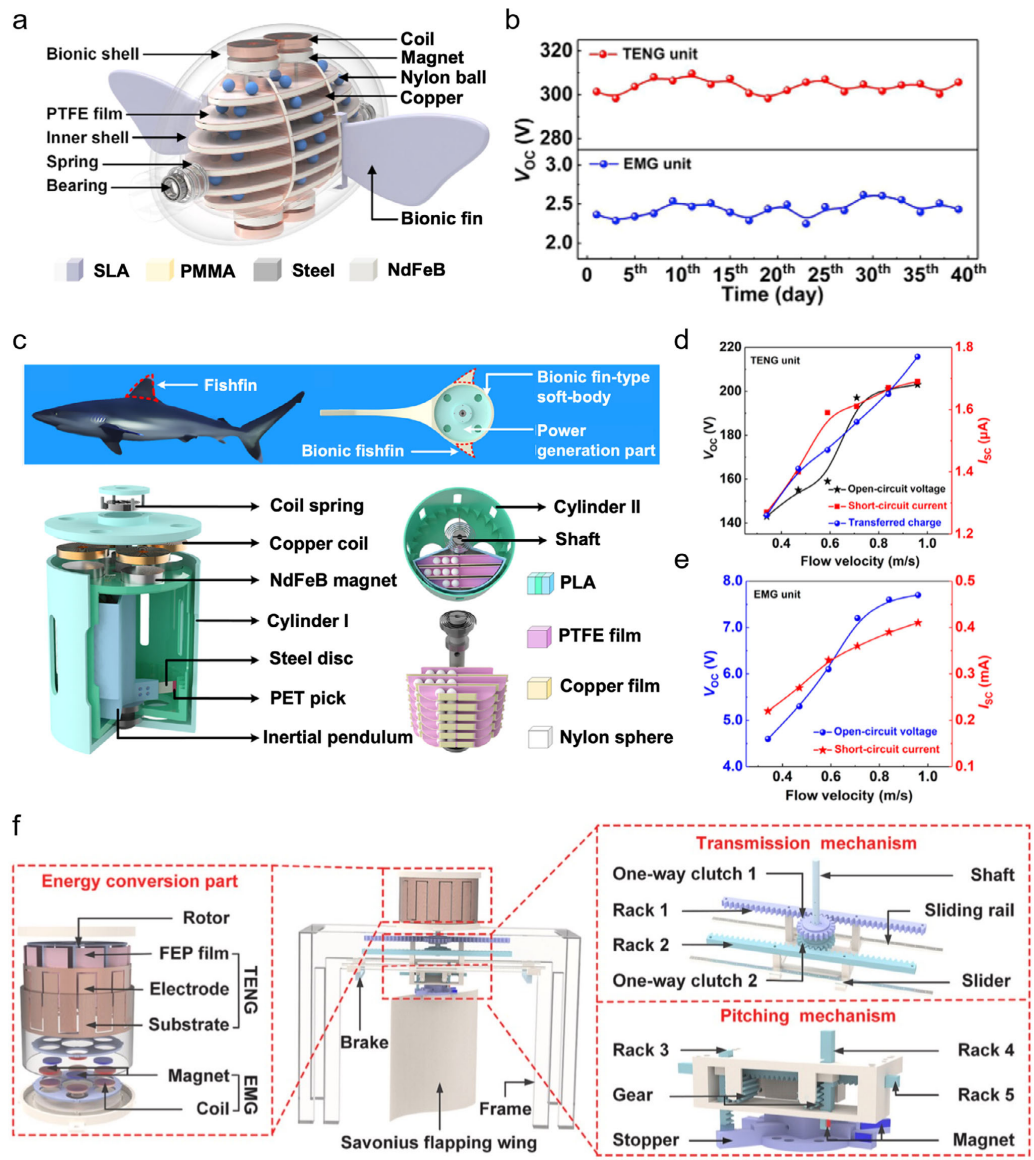
Hybrid HE−TENG for efficient energy harvesting from water flow energy. a) Structure of the BF−TEHG and b) Sealing test of the device after continuous immersion for 40 days. Reproduced with permission [88]. Copyright 2024, ACS Publications. c) Inspiration for the bionic idea of the SF−TEG structure of the power generation part. d) Electrical characteristics of the TENG unit at different flow velocities. e) Electrical characteristics of the EMG unit at different flow velocities. Reproduced with permission [89]. Copyright 2022, ACS Publications. f) Structure and operating states of the SFW−TEHG. Reproduced with permission [90]. Copyright 2024, Elsevier.

Q. Gao et al. proposed a bionic fish−shaped triboelectric−electromagnetic hybrid generator (BF−TEHG) utilizing a two−stage swing mechanism for harvesting water flow energy, as shown in Figure 6a [88]. The BF−TEHG comprises a bionic fish body, TENG, EMG, and a connection unit. The bionic fish structure is designed with a bionic shell, inner shell, and a pair of bionic fins, enabling operation at a minimum flow velocity of 0.24 m s^−1^. The TENG and EMG units are hermetically sealed within the bionic and inner shells, and the generator operates via oscillation induced by each swing. Owing to the double−sealed shell, the TENG unit remains isolated from the aqueous environment, demonstrating excellent water resistance with no degradation in electrical performance even after continuous submersion for 40 days (Figure 6b). At flow velocities of 0.24 and 0.98 m s^−1^, the TENG unit generates outputs of 152 V, 1.4 µA and 306 V, 1.8 µA, respectively, while the EMG unit produces outputs of 0.5 V, 70 µA and 2.5 V, 140 µA. This suggests the potential to achieve environmental adaptability and high energy conversion efficiency through bio−inspired technology. From a perspective of structural synergy, the bionic double sealed shell serves a dual purpose: it effectively isolates the sensitive internal circuitry from harsh aqueous environments while functioning as a unified kinetic receiver. From a structural perspective, the two‐stage swing mechanism amplifies low‐magnitude hydrodynamic forces, which facilitates the simultaneous actuation of the high‐voltage TENG and high‐current EMG units under ultra‐low‐velocity regimes without spatial interference. S. Zhang et al. proposed a soft−bionic−fin structure triboelectric−electromagnetic generator (SF−TEG) with a swing−rotation mechanism, inspired by the vortex effect generated by fish fins, as shown in Figure 6c [89]. This generator consists of a soft body in the shape of a bionic fin and a power generation module incorporating the TENG and EMG. The bluff body, featuring a cross−section shaped like a bionic fin, induces a pronounced vortex effect. Driven by this vortex shedding, the soft body undergoes oscillatory swinging motion, which actuates the internal TENG and EMG to harvest water flow energy. Due to the inertia from swinging, the TENG operates as the internal nylon spheres roll on the PTFE surface, while the EMG operates as the NdFeB magnets rotate. In this system, as shown in Figure 6d,e, at flow velocities ranging from 0.34 to 0.96 m s^−1^, the TENG achieves an open−circuit voltage (*V*_OC_) of 143−203 V and a short−circuit current (*I*_SC_) of 1.27−1.69 µA, while the EMG achieves a *V*_OC_ of 4.6−7.7 V and an *I*_SC_ of 0.22−0.41 mA. This bio−inspired approach enables adaptability and high energy conversion efficiency. However, while optimal energy harvesting performance is achieved under unidirectional water flow, the energy conversion efficiency may diminish when the direction of water flow fluctuates. Consequently, although efficiency may decline in environments with variable flow directions, the structure demonstrates the potential to maximize performance in conditions where the water flow remains constant. In terms of mechanical design, this architecture implements an efficient motion‐conversion mechanism that transforms external, vortex‐induced oscillations into internal continuous rotation. By utilizing a shared kinetic input, the system concurrently actuates the rolling triboelectric spheres and the rotating magnetic rotor, which maximizes volumetric power density within constrained module dimensions. J. Zhang et al. developed the Savonius flapping wing triboelectric‐electromagnetic hybrid generator (SFW‐TEHG) for harvesting hydrokinetic energy from unidirectional low−velocity currents by combining a Savonius airfoil with a flapping wing mechanism, as shown in Figure 6f [90]. The SFW‐TEHG consists of a Savonius flapping wing, a pitching mechanism, a transmission mechanism, and an energy conversion unit including a TENG and EMG. Under a water velocity of 0.21 m s^−1^, the TENG achieves a peak *V*_OC_ of 1.57 kV and a peak *I*_SC_ of 30.94 µA, while the EMG produces a peak *V*_OC_ of 6.24 V and a peak *I*_SC_ of 3.76 mA. To demonstrate practical applicability for agricultural production monitoring, three experiments were conducted using the SFW‐TEHG to power lighting and environmental monitoring devices at a water velocity of 0.31 m s^−1^. The SFW‐TEHG successfully powered 666 LEDs. In Addition, when 10 mF and 4.7 mF capacitors were charged to 3.7 V, the system was able to drive a wireless temperature sensor for environmental monitoring and a wireless light sensor for luminous flux monitoring. This demonstrates the potential for high output in low−velocity water flow environments, enabling diverse applications. However, the requisite modules for operation, such as the pitching and transmission mechanisms, increase mechanical complexity, which could lead to potential long−term operational challenges and elevated fabrication costs. Despite its structural complexity, the SFW‐TEHG demonstrates a highly effective mechanical synergy by employing a ratchet gear rack transmission mechanism to up convert low frequency flapping motions into high frequency rotation. This conversion critically compensates for the inherent inefficiency of the EMG at low speeds. Furthermore, this investigation provides a compelling demonstration of electrical synergy in a real‐world application. By integrating the ultrahigh voltage TENG with the high current EMG, the hybridized system successfully mitigates impedance mismatches. Consequently, it rapidly charges large capacity capacitors (up to 10 mF), thereby enabling the stable, self‐powered operation of practical wireless IoT sensors.

### 3.3 Vibration Energy Harvesting

Conventional reliance on natural resources such as solar, wind, and hydropower is often limited by geographical constraints and weather patterns [91]. In contrast, vibration energy harvesting technology can capture energy from various mechanical and structural vibrations in transportation vehicles, buildings, and industrial equipment, offering wide applicability [92, 93]. Although various methods have been developed to convert vibration energy into electrical energy, TENGs and EMGs are widely utilized due to their high output performance and structural versatility. However, energy harvesters relying on a single transduction mechanism often suffer from limited conversion efficiency, restricting their practical deployment in real−world applications. To overcome these challenges, researchers have explored hybrid energy harvesting systems that integrate TENG and EMG to enhance power output and expand operational frequency bandwidths. This hybrid approach combines the advantages of both technologies, enabling efficient and stable energy conversion from mechanical vibrations over a broad range of frequencies and operational conditions.

**FIGURE 7:**
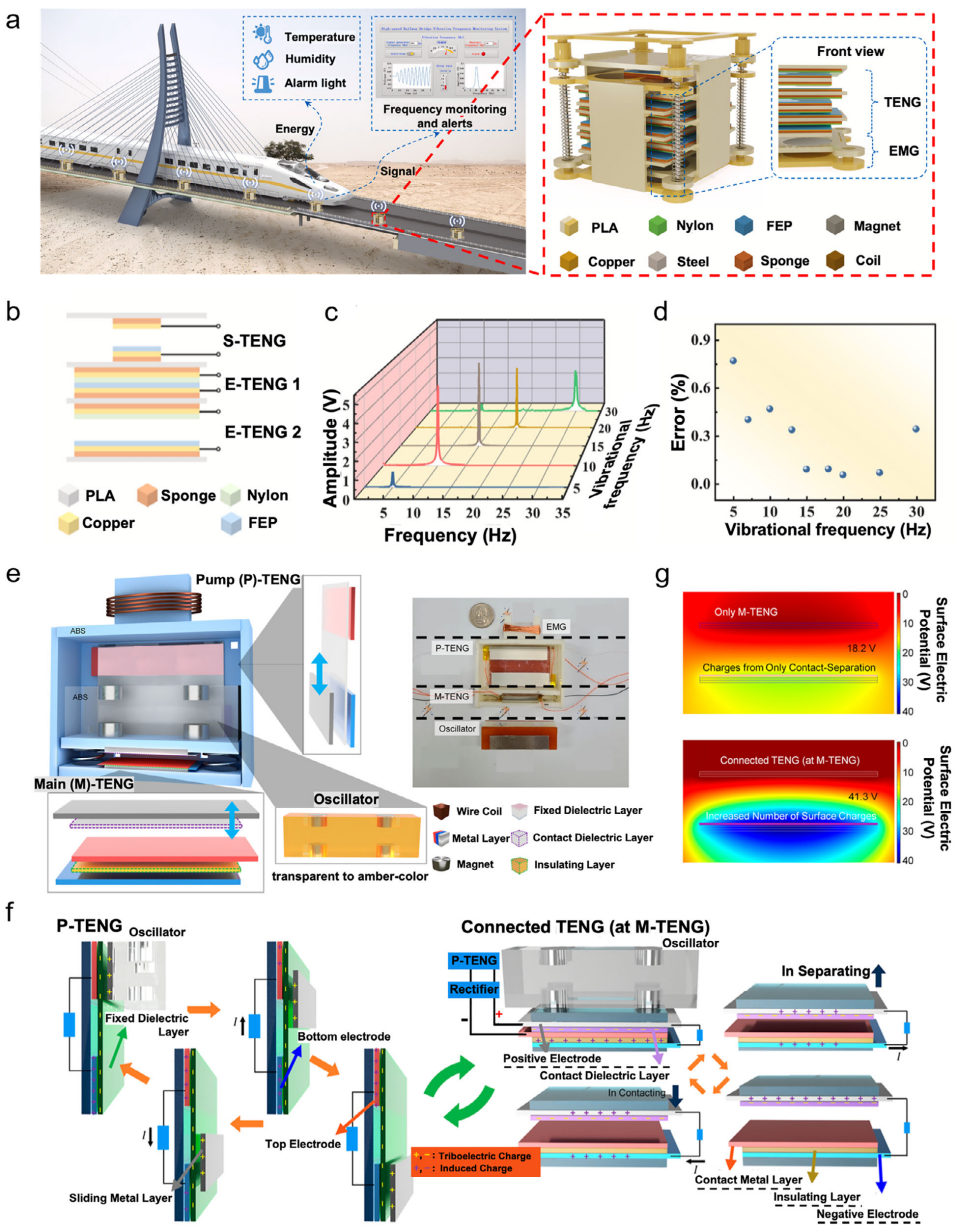
Hybrid HE−TENG for efficient energy harvesting from vibration energy generated in industrial and daily life. a) Schematic oft he M−TENG application for energy harvesting and distributed health monitoring of high−speed railways, and detailed structure of the generator. b) Detailed structure of the TENG units. c) Frequency spectrum plots of the S−TENG after FFT transformation, activated by different frequencies. d) Relative error between the measured frequency and the vibration frequency. Reproduced with permission [94]. Copyright 2023, Elsevier. e) Structural illustration of the oscillating charge pump−based hybrid generator (OCP−HG). f) Operating principles of the P−TENG and the connected TENG. g) FEM simulation result of the surface electric potential at the M−TENG with/without pumping. Reproduced with permission [95]. Copyright 2022, ACS Publications.

J. Shen et al. presented a multilayer triboelectric−electromagnetic hybrid generator (M−TEHG) designed to harvest vibration energy from bridges, ensuring the continuous operation of sensors while accurately capturing the bridge's vibration frequency for early warning systems within the 5−30 Hz range, as shown in Figure 7a [94]. The M−TEHG comprises six layers of TENGs, with its structural configuration illustrated in Figure 7b. The first layer functions as the sensing triboelectric nanogenerator (S−TENG), while the second to sixth layers constitute the energy−harvesting triboelectric nanogenerators (E−TENGs). When high−speed trains pass over bridges, the load induces forced vibrations in the bridge structure. If the load frequency approaches the natural frequency of the bridge, resonance may occur, potentially leading to catastrophic consequences. The *V*_OC_ and *I*_SC_ of the S−TENG were measured in the 5−30 Hz range, with *V*_OC_ ranging from approximately 2 to 14 V and *I*_SC_ from 0.02 to 0.56 µA. The system achieves a peak power density of 2.8 W m^−3^ at 10 Hz. The filtered voltage signals from the S−TENG were subjected to a Fast Fourier Transform (FFT) to extract specific frequencies (Figure 7c). By comparing the frequency obtained via FFT with the input frequency, a relative error of less than 0.9% was achieved in the 5−30 Hz range (Figure 7d). This demonstrates that the S−TENG exhibits excellent performance in monitoring the vibration frequency of high−speed railway bridges. In addition, it is designed to encompass a relatively wide frequency bandwidth (5−30 Hz), extending its applicability to various other vibration monitoring scenarios. From a structural perspective, the M‐TEHG demonstrates spatial integration through integration of sensing and harvesting layers into a unified vertical framework, which enables a single mechanical vibration to simultaneously actuate the S‐TENG, multiple E‐TENGs, and the integrated EMG. Regarding the electrical configuration, this framework establishes a dual‐mode functional integration where the consolidated power output from the hybrid modules sustains the operation of external electronics, whereas the isolated S‐TENG functions as a self‐powered frequency monitor. I. Kim et al. developed an oscillating charge pump−based hybrid generator (OCP−HG) to enhance the output of TENGs by utilizing a method that pumps charges onto the contact metal surface of the TENG [95]. The OCP−HG incorporates two TENG units a cuboid−type freestanding TENG and a simple contact−separation−based double−electrode TENG hybridized with an EMG. This structural design facilitates efficient operation under vertical vibrations. As shown in Figure 7e, the OCP−HG is composed of a pump−TENG (P−TENG), a main−TENG (M−TENG), an oscillator, and an EMG. The operational mechanism of the interconnected M−TENG and P−TENG is governed by the reciprocating motion of the oscillator, as illustrated in Figure 7f. As the P−TENG operates, it generates an AC signal through the two electrodes, which is supplied to the M−TENG to pump charges. This process increases the surface charge density, thereby enhancing the potential difference and improving the output power. According to the Finite Element Method (FEM) analysis in Figure 7g, when charge pumping is applied, the potential difference between the two electrodes of the TENG increases to 41.3 V, compared to 18.2 V in the absence of charge pumping, confirming a threefold increase in charge accumulation. At an input frequency of 6 Hz, the M−TENG, P−TENG, and the connected TENG exhibit a *V*_OC_ of 24.23, 52.23, and 85.73 V, with *I*_SC_ values of 2.249, 1.10, and 9.168 µA, respectively. For the EMG, the *I*_SC_ is 1.183 mA, and the *V*_OC_ is 504 mV. This approach eliminates the need for complex switching circuits by utilizing a timing synchronization method, where the P−TENG acts as a charge pump for the M−TENG, allowing it to accumulate greater charge and generate higher voltage and current. Such results suggest a new direction for TENG systems, where multiple TENGs work synergistically to produce a cumulative effect that significantly enhances overall performance compared to the traditional standalone TENGs. From a structural perspective, the implementation of a shared central oscillator facilitates structural integration by synchronizing the reciprocating motions of the P‐TENG, M‐TENG, and EMG components along a single vertical axis, which obviates the requirement for complex mechanical switching mechanisms. Regarding the electrical architecture, the system couples the complementary impedance profiles of the TENG and EMG units while incorporating an internal charge‐management mechanism, where the P‐TENG facilitates synchronous charge‐pumping into the M‐TENG. This switchless charge‐pumping approach demonstrates how internal charge regulation and multi‐mechanism hybridization are integrated to enhance energy‐harvesting performance under vibration stimuli.

### 3.4 Human Motion Energy harvesting

With the rapid advancement of the IoT, wireless technologies, and portable electronics, widely distributed devices play crucial roles in areas such as environmental monitoring, motion sensing, and healthcare [96]. Currently, these devices predominantly rely on electrochemical batteries, which possess limited energy capacity and require frequent recharging or replacement, leading to elevated maintenance costs and environmental concerns [97]. To address these power limitations, researchers have explored biomechanical energy harvesters that can convert human motion into usable electrical energy. Given that the motion of the human body is characterized by low frequency and irregular amplitude, TENGs have emerged as a promising technology for this application.

**FIGURE 8:**
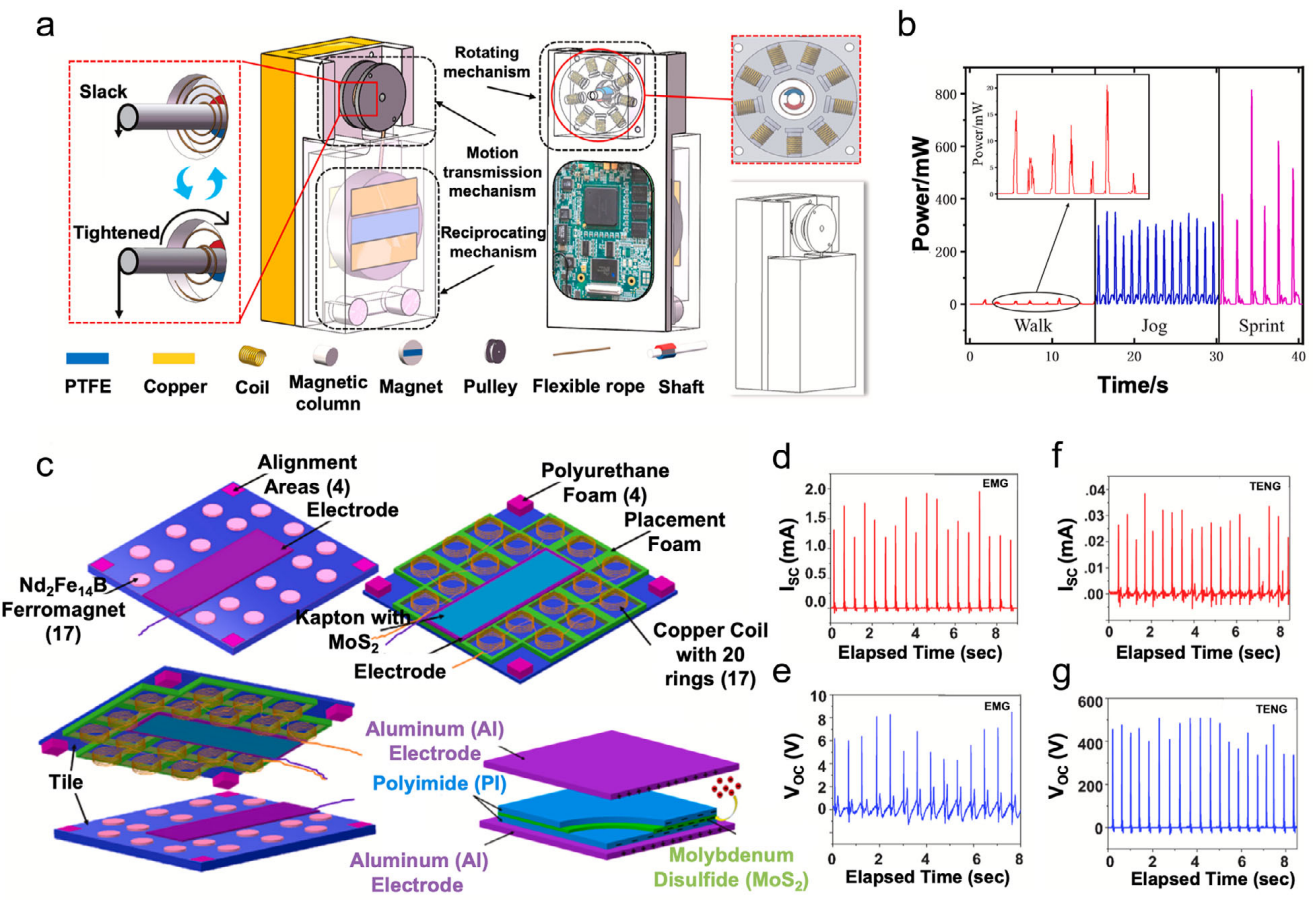
Hybrid HE−TENG for efficient energy harvesting from human motion. a) Schematic diagram of the ET−HEH. b) Influence of the human motion states on the output performance of the ET−HEH. Reproduced with permission [98]. Copyright 2023, Elsevier. c) Internal structure of the tile and vertical contact−separation mode TENG with a MoS2 layer. TENG and EMG Individual Performance in walking conditions d) EMG Short Circuit Current, e) EMG Open Circuit Voltage, f) TENG Short Circuit Current, g) TENG Open Circuit Voltage. Reproduced with permission [99]. Copyright 2020, Elsevier.

S. Bai et al. proposed a high power electromagnetic−triboelectric hybrid energy harvester (ET−HEH) featuring a broad frequency response bandwidth and an efficient energy transfer strategy [98]. As shown in Figure 8a, the system operates by utilizing scroll springs and pulleys within a rotational electromagnetic generator (REMG) to convert reciprocating vibration into high−speed rotation. In addition, a vibration triboelectric nanogenerator (VTENG) is integrated into the reciprocating vibration structure to enhance energy harvesting efficiency. The performance of the ET−HEH was evaluated under diverse motion states to assess its practical applicability. The measurements were conducted in three distinct motion states: walking, jogging, and sprinting at 4, 12, and 20 km h^−1^, respectively (Figure 8b). During walking, the output reached 20 mW; it stabilized around 300 mW during jogging and achieved a peak output of 800 mW with a stable output between 350–400 mW during sprinting. By harvesting energy generated from human motion, the ET−HEH successfully charged a Bluetooth headset and a smart band with a rated power of 400 mW, demonstrating the significant potential of the proposed harvester for the development of self−powered human monitoring sensors and portable electronic devices. From a structural perspective, the ET‐HEH utilizes scroll springs and pulleys to achieve geometric frequency up‐conversion, which transforms irregular, low‐frequency reciprocating human motion into high‐speed continuous rotation for the REMG. Simultaneously, architecture utilizes residual linear displacement to actuate the VTENG unit. Regarding electrical integration, the consolidation of the high‐voltage VTENG and high‐current REMG outputs facilitates a stable power supply, which reaches 400 mW during sprinting. This configuration addresses the inherent impedance mismatches of individual modules, enabling the direct operation of commercial wearable electronics. E. Islam et al. presented a hybridized triboelectric and electromagnetic energy tile designed for high voltage and current generation [99]. Figure 8c shows that the tile consists of a simplified configuration of coils and magnets, incorporating a multilayer system that utilizes Kapton (polyimide) as the triboelectric material, MoS_2_ (molybdenum disulfide) as the electron acceptor, and positively charged aluminum (Al) as electrodes. This study introduces a hybrid technology of TENG and EMG integrated into a floor tile structure to harness previously unutilized human footstep energy for cost−effective and sustainable energy generation. At a walking frequency of 2 Hz (120 BPM), with a 63.5 kg load and a foot−to−tile distance of 0.076 m, the output results for the TENG and EMG indicate that the *I*_SC_ of the EMG varies between 1.25 and 2 mA, with the *V*_OC_ ranging from a maximum of 8 V to a minimum of 4 V (Figure 8d,e). The *I*_SC_ of the TENG fluctuates between 20 and 38 µA, with the *V*_OC_ achieving a maximum of 500 V and a minimum of 350 V (Figure 8f,g). The hybrid system demonstrated a 25−fold increase in open−circuit voltage and 20% higher power output compared to commercially available energy−harvesting floor tiles such as Pavegen. The harvested energy can be stored in capacitors or utilized for real−time applications like LED lighting and sensors. This dual functionality of energy harvesting and real−time sensing, in particularly for applications in smart cities, renders it ideal for environments such as transportation hubs, gyms, and streets. However, the system achieves optimal power output when the human movement frequency falls within a specific range; as the frequency deviates from this optimum, the power generation efficiency diminishes. Therefore, while the system holds great potential for areas with consistent pedestrian flow, it may face challenges in environments characterized by highly variable movement frequencies. From a structural standpoint, the floor tile architecture demonstrates optimal spatial synergy by seamlessly integrating the TENG dielectric layers directly with the EMG coil magnet arrays. This configuration facilitates the simultaneous actuation of both energy conversion mechanisms by a single footstep within a low‐profile architecture. Regarding electrical integration, the system combines the high voltage of the TENG (up to 500 V) with the current output of the EMG (up to 2 mA), which provides a reliable and scalable power source for distributed smart city sensors. These findings establish that structural and electrical hybridization represents an essential framework for addressing the stochastic, low‐frequency nature of human biomechanics, thereby enabling the development of autonomous wearable electronics and intelligent infrastructure.

### 3.5 Comparative Analysis of HE−TENG Designs Across Energy Sources

As discussed in the preceding sections, HE‐TENGs exhibit unique structural adaptations tailored to the specific physical characteristics of diverse mechanical energy sources. Table 2 comprehensively delineates the driving mechanisms, device dimensions, and electrical output profiles of the representative investigations reviewed herein. A fundamental commonality across all HE‐TENG architectures is the objective of mitigating the impedance mismatch between the low frequency and high voltage TENG and the high frequency and high current EMG to establish robust electrical synergy. Nevertheless, distinct variations emerge in their primary mechanical mechanisms and spatial configurations, heavily contingent upon the target energy source. For wind energy harvesting, which is characterized by unidirectional and relatively continuous flows, rotational mechanisms utilizing turbine blades are predominantly employed to maximize aerodynamic efficiency. Conversely, for water waves and fluid flows, which exhibit omnidirectional and ultra‐low frequency motions, pendulum or swing oscillation mechanisms, often incorporating bionic fins or liquid guided structures, are essential for effectively capturing irregular aquatic fluctuations. Concurrently, energy harvesters designed for vibration and human biomechanical motion face stringent constraints regarding spatial footprint and motion continuity. To achieve optimal efficiency within compact dimensions, these systems frequently adopt vertical contact separation multilayer architectures or frequency up‐conversion mechanisms that geometrically translate low frequency linear vibrations into high‐speed continuous rotations. These structural disparities directly dictate the electrical output profiles. While wind based HE‐TENGs tend to generate a relatively stable average power output, vibration and biomechanical systems are predominantly optimized to maximize instantaneous V_OC_ and I_SC_, facilitating the rapid charging of energy storage capacitors. Ultimately, the development of a highly efficient HE‐TENG system must transcend the mere physical integration of two distinct generators. It strictly necessitates a synergistic mechanical design that is precisely tailored to the frequency bandwidth, directionality, and spatial constraints of the targeted ambient energy source.

**TABLE 2:** Comprehensive performance comparison of recently reported HE−TENGs for diverse environmental energy scavenging applications.

Energy source	Refs.	Mechanical environment	Dimensions	Electric characterization EMG	Electric characterization TENG	Electric characterization HE‐TENG	Mechanical structure	Synergistic mechanisms (Mechanical & Electrical)
Wind	[73]	4–9 m/s, 50–300 rpm (Continuous, unidirectional)	Φ 200 mm × 170 mm	V_OC_ = 22 V, I_SC_ = 5.0 mA, Power = 14.0 mW, @ 2.2 kΩ	V_OC_ = 390 V, I_SC_ = 12 µA, Power = 5.2 mW, @ 50 MΩ	ND	Rotational	Mechanical synergy (Frequency‐dependent mechanical switching)
	[74]	2.4–5 m/s (Irregular and stochastic)	ND	V_OC_ = 3.95 V, I_SC_ = 8.74 mA, Power = 14.0 mW, @ 500 Ω	V_OC_ ≈ 120 V, I_SC_ ≈ 1.5 µA, Power = 0.23 mW, @ 200 MΩ	ND	Rotational	Electrical Synergy (voltage and current‐stabilizing circuits)
	[75]	2.4–5 m/s (Continuous low‐velocity)	200 mm × 200 mm × 150 mm	V_OC_ = 74 V, I_SC_ = 13.1 mA	V_OC_ = 80 V, I_SC_ = 1.0 µA	V_OC_ = 154 V, I_SC_ = 13.1 mA, Total_Power = 45.5 mW	Rotational	Mechanical synergy (Frequency dependent TENG mode transition)
Water wave	[84]	0.2–1.0 Hz (Ultra‐low frequency and Omnidirectional)	ND	V_OC_ = 2.30 V, I_SC_ = 10.43 mA, Power density = 148.24 W/m^3^, @ 500 Ω	V_OC_ = 230.25 V, I_SC_ = 1.34 µA, Power density = 13.77 W/m^3^, @ 9000 MΩ	ND	Spherical / Rolling Ball	Mechanical synergy (Integrated hybrid structure)
	[85]	0.67–1.47 Hz (Omnidirectional and highly stochastic)	ND	Power = 10.1 mW, @ 4 kΩ	V_avg_ = 168.08 V, I_avg_ = 2.03 µA, Power = 101.5 µW, @ 50 MΩ	Current density = 4.85 µA/cm^3^, Power density = 7.25 µW/cm^3^	Liquid‐Solid Interfaced	Mechanical synergy (Mechanically eliminated rigid solid friction)
	[86]	0.1 Hz (Ultra‐low frequency)	≈ Φ 100 mm	V_OC_ = 2.9 V, I_SC_ = 11. 9 mA, Power = 3.5 mW, @ 300 Ω	V_OC_ = 640 V, I_SC_ = 4.57 µA, Power = 1.29 mW, @ 150 MΩ	Power = 4.8 mW, Power density = 10.16 mW/m^3^	Swing Pendulum	Mechanical synergy (Mechanically induced elastic soft contact to minimize resistance)
Water flow	[88]	0.24–0.98 m/s (Continuous and ultra‐low velocity)	≈ 140 mm × 64 mm × 140 mm	V_OC_ = 2.5 V, I_SC_ = 140 µA, Power = 0.34 mW, @ 9 kΩ	V_OC_ = 306 V, I_SC_ = 1.8 µA, Power = 0.55 mW, @ 500 MΩ	ND	Bio‐inspired / Rolling structure	Mechanical synergy (Dual generator operation)
	[89]	0.34–0.96 m/s (Continuous and low velocity)	ND	V_OC_ = 7.7 V, I_SC_ = 0.41 mA, Power = 0.403 mW, @ ≈ 1 kΩ	V_OC_ = 203 V, I_SC_ = 1.69 µA, Power = 0.064 mW, @ ≈ 400 MΩ	ND	Bio‐inspired / Swing structure	Mechanical synergy (Swing to rotation mechanism)
	[90]	0.21–0.64 m/s (Continuous and ultra‐low velocity)	≈ 250 mm × 250 mm × 360 mm	V_OC_ = 10.68 V, I_SC_ = 5.43 mA, Power = 9.36 mW, @ 1 kΩ	V_OC_ = 2.20 kV, I_SC_ = 48.70 µA, Power = 37.28 mW, @ 100 MΩ	ND	Bio‐inspired / Rotation structure	Mechanical synergy (Dual generator operation)
Vibration energy	[94]	5–30 Hz (Low‐frequency and continuous)	100 mm x 100 mm x 100 mm	V_OC_ = 0.33 V, I_SC_ = 0.94 mA, Power = 0.19 mW, @ 1 kΩ	V_OC_ = 80 V, I_SC_ = 22.5 µA, Power = 0.68 mW, @ 10 MΩ	Power density = 2.8 W/m^3^	Multilayer structure	Electrical synergy (Fater charging using BUCK circuit)
	[95]	2–10 Hz (Vertical and impact‐driven)	76 mm x 26 mm x 76 mm	V_OC_ = 0.504 V, I_SC_ = 1.183 mA, Power = 66.4 µW, @ 300 Ω	V_OC_ = 85.73 V, I_SC_ = 9.168 µA, Power = 490 µW, @ 40 MΩ	ND	Multilayer / Spring‐Oscillator structure	Electrical synergy (Electrical synergy via charge pumping)
Human motion	[98]	1–15 Hz (Irregular and reciprocating)	6.93 cm^3^	ND	ND	V_OC_ ≈ 10 V, I_OC_ ≈ 14 mA, Power = 450 mW	Vibration to rotation structure	Mechanical synergy (Low‐frequency vibration up‐conversion)
	[99]	60–120 BPM, 1–2 Hz (Vertical and impact‐driven)	0.305×0.305 m^2^	V_OC_ = 8, I_SC_ = 2 mA	V_OC_ = 500 V, I_SC_ = 38 µA	V_OC_ = 1200 V, I_SC_ = 5 mA, Power = 6 W	Floor tile structure	Mechanical synergy (Shared vertical impact absorption in a stacked TENG‐EMG tile)

## 4 Conclusion and Perspective

This review has systematically analyzed the operating principles, structural configurations, and diverse applications of the HE−TENG system through an integrated framework that accounts for both mechanical and electrical coupling. Bridging the inherent mismatch between stochastic ambient energy and the operational requirements of standalone harvesters enables this hybrid approach to facilitate broadband energy harvesting with enhanced power density. The distinct electrical characteristics of these respective generators, specifically the high output voltage of TENGs at low frequencies and the high output current of EMGs at high frequencies, ensure a robust broadband response across a wide mechanical spectrum. Consequently, this hybridized architecture achieves a superior power density compared to isolated discrete units. This integration strategy has been extensively demonstrated as a robust framework for harvesting energy from stochastic ambient sources, which contain wind, water waves, and hydrokinetic flows. While advanced architectures, such as bio−inspired structures, magnetic levitation mechanisms, and solid−liquid interfaces, have mitigated issues related to high cut−in velocities and material abrasion, the transition from laboratory prototypes to reliable industrial applications requires resolving several remaining technical bottlenecks. First, enhancing long‐term mechanical durability is crucial. Despite recent progress, the inevitable material wear in contact‐mode TENGs and the fatigue of mechanical transmission units (e.g., gears and springs) remain primary obstacles in harsh environments. Future research should focus on the development of wear‐resistant polymers and the optimization of non‐contact or soft‐contact interfaces to ensure structural integrity over millions of cycles. Second, the dimensional scaling effects in hybrid architecture constrain the miniaturization process. Since TENG output follows area‐scaling laws while EMG performance scales volumetrically, downscaling leads to a disproportionate reduction in power density. Addressing this challenge requires integrated structural designs, such as dual‐purpose components where a single element functions as both a magnetic source and a dielectric layer, which maximizes volumetric power density within a limited volume. Third, the development of specialized power management integrated circuits (PMICs) is essential. Effective power combining of TENGs and EMGs requires active impedance matching that operates with low power consumption to mitigate inherent impedance mismatches. Future research should emphasize the transition toward self‐adaptive systems by incorporating machine learning‐assisted design and monolithic energy storage modules, ultimately realizing truly autonomous, maintenance‐free IoT networks.

## Conflicts of Interest

The authors declare no conflicts of interest.

## Data Availability

The authors have nothing to report.
